# Evaluating the potential of third generation metagenomic sequencing for the detection of BRD pathogens and genetic determinants of antimicrobial resistance in chronically ill feedlot cattle

**DOI:** 10.1186/s12917-022-03269-6

**Published:** 2022-06-02

**Authors:** Claire N. Freeman, Emily K. Herman, Jennifer Abi Younes, Dana E. Ramsay, Nathan Erikson, Paul Stothard, Matthew G. Links, Simon J. G. Otto, Cheryl Waldner

**Affiliations:** 1 Department of Large Animal Clinical Sciences, Western College of Veterinary Medicine, University of Saskatchewan, 52 Campus Drive, Saskatoon, SK S7N 5B4 Canada; 2 Department of Agricultural, Food, and Nutritional Science, Faculty of Agricultural, Life, and Environmental Sciences, University of Alberta, 2-31 General Services Building, Edmonton, AB T6G 2H1 Canada; 3 Department of Animal and Poultry Science, University of Saskatchewan, 51 Campus Drive, Saskatoon, SK S7N 5A8 Canada; 4 Department of Computer Science, University of Saskatchewan, 110 Science Place, Saskatoon, Saskatchewan S7N 5C9 Canada; 5 HEAT-AMR (Human-Environment-Animal Transdisciplinary AMR) Research Group, School of Public Health, University of Alberta, 11322 89 Ave NW, Edmonton, AB T6G 2G7 Canada

## Abstract

**Background:**

Bovine respiratory disease (BRD) is an important cause of morbidity and mortality and is responsible for most of the injectable antimicrobial use in the feedlot industry. Traditional bacterial culture can be used to diagnose BRD by confirming the presence of causative pathogens and to support antimicrobial selection. However, given that bacterial culture takes up to a week and early intervention is critical for treatment success, culture has limited utility for informing rapid therapeutic decision-making. In contrast, metagenomic sequencing has the potential to quickly resolve all nucleic acid in a sample, including pathogen biomarkers and antimicrobial resistance genes. In particular, third-generation Oxford Nanopore Technology sequencing platforms provide long reads and access to raw sequencing data in real-time as it is produced, thereby reducing the time from sample collection to diagnostic answer. The purpose of this study was to compare the performance of nanopore metagenomic sequencing to traditional culture and sensitivity methods as applied to nasopharyngeal samples from segregated groups of chronically ill feedlot cattle, previously treated with antimicrobials for nonresponsive pneumonia or lameness.

**Results:**

BRD pathogens were isolated from most samples and a variety of different resistance profiles were observed across isolates. The sequencing data indicated the samples were dominated by *Moraxella bovoculi, Mannheimia haemolytica, Mycoplasma dispar,* and *Pasteurella multocida,* and included a wide range of antimicrobial resistance genes (ARGs), encoding resistance for up to seven classes of antimicrobials. Genes conferring resistance to beta-lactams were the most commonly detected, while the *tetH* gene was detected in the most samples overall. Metagenomic sequencing detected the BRD pathogens of interest more often than did culture, but there was limited concordance between phenotypic resistance to antimicrobials and the presence of relevant ARGs.

**Conclusions:**

Metagenomic sequencing can reduce the time from sampling to results, detect pathogens missed by bacterial culture, and identify genetically encoded determinants of resistance. Increasing sequencing coverage of target organisms will be an essential component of improving the reliability of this technology, such that it can be better used for the surveillance of pathogens of interest, genetic determinants of resistance, and to inform diagnostic decisions.

**Supplementary Information:**

The online version contains supplementary material available at 10.1186/s12917-022-03269-6.

## Background

Bovine respiratory disease (BRD) is an important cause of morbidity and mortality and is responsible for most of the injectable antimicrobial use in the feedlot industry [1]. BRD is a complex, multifactorial disease linked to a combination of viral and bacterial pathogens together with management and environmental factors impacting the host immune response [2]. Laboratory diagnostics are critical to inform appropriate antimicrobial use for disease management and therapy and to guide vaccination recommendations. Traditional bacterial culture with or without antimicrobial susceptibility testing (AST) can be used to confirm the presence of causative pathogens and to support antimicrobial selection. However, given that culture-based approaches can take up to a week to finalize and early intervention is critical to BRD treatment success [3], these approaches have limited utility for informing rapid therapeutic decisions. Recent WHO prescribing guidelines recommend basing all prescriptions for livestock on diagnostic test data [4]. 

In contrast to classical culture-based microbiology methods, molecular techniques including quantitative polymerase chain reaction (qPCR), whole genome sequencing (WGS), and metagenomic sequencing are gaining traction in diagnostic laboratories due to their comparatively faster speeds and potential for increased sensitivity. WGS generally requires that bacterial and viral pathogens are first isolated and cultured prior to nucleic acid extraction and sequencing, but can produce high resolution genomic information for serotyping, outbreak surveillance, and outbreak management [5–7]. qPCR and metagenomics differ from WGS in that they can both use DNA extracted directly from clinical samples, bypassing time-consuming culture-based steps. qPCR is already widely used in BRD diagnostics, and even exists in the form of commercially available kits (Pneumo4, DNA Diagnostic A/S, Risskov, Denmark). More recently, qPCR has been used to quantify the relative proportions of antimicrobial resistance genes (ARGs) in the nasopharyngeal microbiota of Canadian feedlot cattle [8, 9].

Metagenomics refers to either 1) amplicon sequencing, wherein a single conserved microbial gene is amplified and sequenced; or 2) shotgun metagenomics, wherein all DNA extracted from a sample is made available for sequencing. Although amplicon sequencing can offer improved species detection over shotgun metagenomics, it is not suitable for providing genetic information outside of taxonomic composition and relative abundance. Due to its untargeted nature, metagenomic sequencing has the potential to reveal anything encoded by nucleic acids present in the sample, including pathogen biomarkers, ARGs, and virulence genes [10]. In contrast to qPCR, the use of metagenomics in diagnostics has only recently been explored [11]. An examination of the diagnostic potential of sequencing technologies for BRD must evaluate its performance in tandem with existing gold standard methods, including bacterial culture and AST.

Concordance between culture, AST and WGS sequence data varies depending on the bacterial species, the tested antimicrobials, and the ARGs under investigation. For example, phenotypic resistance correlated highly (99%) with the presence of known resistance determinants in isolates of nontyphoidal *Salmonella* from clinical and retail meat samples [12]. In contrast, the concordance between genotype and phenotype for antimicrobial-resistant *Mannheimia haemolytica* from cattle is reportedly lower, particularly for resistance to tilmicosin, tulathromycin, and florfenicol, which was largely as a result of ARG-containing integrative and conjugative elements (ICE) [13]. Substantial genotype–phenotype discordance was likewise noted in a WGS study involving BRD-associated isolates (*M. haemolytica, Pasteurella multocida* and *Histophilus somni*) from beef and dairy calves [14].

Concordance between culture and metagenomic sequencing is decidedly less well characterized and faces many additional obstacles, including variable sequence quality, the absence of data related to transcriptional activity, and the presence of abundant host DNA [15]. In one study that compared 16S rRNA amplicon sequence data to conventional culture results from clinical samples, the authors reported a 91.8% and 52.8% concordance rate for culture-positive and culture-negative specimens, respectively [16]. Other studies have reported that measures of genotype–phenotype concordance are highly variable for the same sample depending on the bioinformatic analysis used [17].

With the exception of select metagenomic surveys that consider BRD-associated viral pathogens [18, 19], most BRD studies that employ metagenomic or whole-genome sequencing have used second-generation, short-read sequencing on Illumina, Ion Torrent, and Roche 454 sequencing platforms [20, 21]. These platforms generally offer high sequence quality but have otherwise limited practicality for rapid diagnostics due to their lengthy library preparation protocols and the inaccessibility of data until after the sequencing run has finalized. In contrast, third-generation Oxford Nanopore Technology (ONT) platforms provide long reads and access to raw sequencing data in real-time as it is produced; this feature allows for bioinformatic analyses to be run simultaneously, including the identification of pathogens and ARGs. The feasibility of using an ONT device to produce sequencing data, assemble reads, and annotate the genomes of two *M. haemolytica* strains from pneumonic cattle was explored in a recent study [22]. The authors highlight the potential of nanopore technology to evaluate AMR, given that ARGs were identified from assemblies constructed with relatively few (> 5400) ultra-long reads and corresponded in most cases to phenotypic resistance.

Feedlot cattle that receive repeated treatments for BRD and fail to respond are generally relocated to designated “chronic pens” [23]. These animals are more likely than the general feedlot population to have been administered multiple classes of antimicrobials as part of their ongoing medical management [23]. The purpose of this study was to compare the diagnostic performance of nanopore metagenomic sequencing to traditional culture and sensitivity methods when applied to clinical nasopharyngeal samples from segregated groups of chronically ill feedlot cattle, primarily afflicted with nonresponsive pneumonia or lameness. The following proof of concept study to evaluate the use of direct DNA metagenomic sequencing to detect BRD pathogens and their resistance determinants thus targeted this subpopulation where both were expected to be present in greater relative proportions.

## Results

Sequencing yielded 73.99 Gb in total (average 12.33 ± 2.79 per run) and produced 57.32 million reads (average 9.55 ± 2.03 per run). After quality filtering, each sample had an average of 1.93 Gb of data (± 0.67 Gb), while after host filtering, an average of 115.4 million bases remained per sample (full details in Supplementary Table 1, Additional File 1). Data have been deposited with the Short Read Archive (BioProject: PRJNA809384). The mean quality score of the post quality filtered reads was 13.7 ± 0.3. In general, the resulting sequences were mostly derived from host biomass, as an average of 94% of each sample was classified as *Bos taurus* by the bioinformatic pipeline*.*

### Culture and antimicrobial susceptibility

Target BRD pathogens were isolated from 20 of 25 samples; *P. multocida* (*n* = 16) and *M. bovis* (*n* = 12) were the most commonly detected organisms. *M. haemolytica* was present in comparatively low numbers (*n* = 2), and *H. somni* was not isolated from any sample. Co-isolation of pathogens was common (*n* = 10). All recovered *M. haemolytica* and *P. multocida* isolates were susceptible to ceftiofur, danofloxacin, enrofloxacin and florfenicol. The MICs for all tested antimicrobial drugs are reported for each isolate in Table 1.

**Table 1:** Minimum inhibitory concentrations for isolates recovered in this study

Sample No	BRD Pathogen	Culture Result	AMP	TIO	CLIN*	DANO	ENRO	FLOR	GAM	GEN*	NEO*	PEN	SPECT	SUL*	TET	TIAM*	TILD	TILM	TRIMS*	TULA	TYL*	CTET	OTET
1	*M. bovis*		-	-	-	-	0.12	4	128	-	-	16	-	-	-	-	256	512	-	64	256	4	2
2	*M. bovis*		-	-	-	-	0.12	4	64	-	-	16	-	-	-	-	256	512	-	8	32	8	4
3	*P. multocida*	Few	0.25	0.25	32	0.12	0.12	0.50	16	4	64	0.12	128	512	8.0	64	32	32	2	128	64	-	-
5	*M. bovis*		-	-	-	-	8.00	8	512	-	-	16	-	-	-	-	256	512	-	512	256	4	4
5	*P. multocida*	1 +	0.25	0.25	32	0.12	0.12	0.50	1	4	8	0.12	32	256	0.5	32	1	4	2	8	32	-	-
6	*P. multocida*	2 +	0.50	0.25	32	0.12	0.12	0.50	2	4	64	1.00	32	512	8.0	32	2	16	4	8	64	-	-
7	*M. bovis*		-	-	-	-	4.00	4	256	-	-	16	-	-	-	-	256	512	-	512	32	16	8
7	*P. multocida*	1 +	0.25	0.25	16	0.12	0.12	0.50	1	1	4	0.25	32	512	0.5	8	1	2	2	8	16	-	-
8	*M. bovis*		-	-	-	-	4.00	8	512	-	-	16	-	-	-	-	256	512	-	512	256	8	4
8	*P. multocida*	1 +	0.25	0.25	32	0.12	0.12	0.50	1	2	8	0.12	32	256	0.5	16	1	4	2	8	32	-	-
9	*M. bovis*		-	-	-	-	4.00	4	512	-	-	16	-	-	-	-	256	512	-	512	256	8	4
9	*P. multocida*	4 +	0.50	0.25	32	0.12	0.12	0.50	2	4	64	2.00	32	512	4.0	64	2	16	4	8	64	-	-
10	*M. bovis*		-	-	-	-	0.12	2	32	-	-	16	-	-	-	-	128	256	-	8	32	8	4
10	*P. multocida*	1 +	0.25	0.25	32	0.12	0.12	0.50	1	4	16	0.25	32	512	0.5	32	1	4	2	8	32	-	-
11	*M. bovis*		-	-	-	-	2.00	2	512	-	-	16	-	-	-	-	256	512	-	2	256	1	1
11	*P. multocida*	1 +	0.25	0.25	32	0.12	0.12	0.50	1	4	8	0.12	32	256	0.5	32	1	8	2	8	64	-	-
12	*M. bovis*		-	-	-	-	4.00	2	512	-	-	16	-	-	-	-	256	128	-	512	256	4	2
12	*P. multocida*	3 +	4.00	0.25	32	0.12	0.12	0.25	1	4	64	16.0	32	512	4.0	64	2	8	4	8	64	-	-
13	*M. bovis*		-	-	-	-	0.50	4	512	-	-	16	-	-	-	-	256	512	-	512	64	4	2
14	*M. bovis*		-	-	-	-	0.12	4	128	-	-	16	-	-	-	-	128	256	-	64	64	4	2
14	*P. multocida*	1 +	16.0	0.25	32	0.12	0.12	0.50	2	4	64	16.0	32	512	4.0	64	2	16	4	8	64	-	-
17	*M. haemolytica*	1 +	0.25	0.25	8	0.12	0.12	1.00	16	2	4	0.25	32	512	0.5	16	1	16	2	64	64	-	-
18	*P. multocida*	2 +	0.25	0.25	32	0.12	0.12	0.25	1	2	64	0.50	32	512	4.0	32	2	8	4	8	64	-	-
19	*P. multocida*	4 +	0.25	0.25	32	0.12	0.12	0.50	1	2	8	0.25	16	256	0.5	32	1	8	2	8	64	-	-
20	*P. multocida*	1 +	4.00	0.25	32	0.12	0.12	0.50	2	4	64	8.0	32	512	8.0	64	2	16	4	8	64	-	-
23	*P. multocida*	1 +	0.25	0.25	32	0.12	0.12	0.50	1	2	8	0.25	32	256	0.5	32	1	8	2	8	64	-	-
24	*M. haemolytica*	1 +	0.25	0.25	8	0.12	0.12	0.50	1	1	4	0.12	32	256	0.5	16	1	8	2	8	64	-	-
24	*P. multocida*	1 +	0.25	0.25	32	0.12	0.12	0.50	1	2	8	0.12	32	256	0.5	32	2	8	2	8	64	-	-
25	*M. bovis*		-	-	-	-	0.12	4	512	-	-	16	-	-	-	-	256	512	-	512	256	8	4
25	*P. multocida*	1 +	0.50	0.25	32	0.12	0.12	0.50	1	2	8	0.12	32	256	0.5	32	1	8	2	8	64	-	-

Samples were screened for the presence of BRD pathogens and recovered isolates were tested for sensitivity to antimicrobials via serial broth microdilution using the BOPO7F panel. MICs for each antimicrobial were compared against CLSI breakpoints (full details in Supplementary Table 2, Additional File 2. Table entries with a dash indicate an isolate was not tested against a certain antimicrobial. Antimicrobials without established breakpoints for the target organisms are indicated with an asterisk

### Bacterial and ARG abundance in the sequence data

**Fig. 1:**
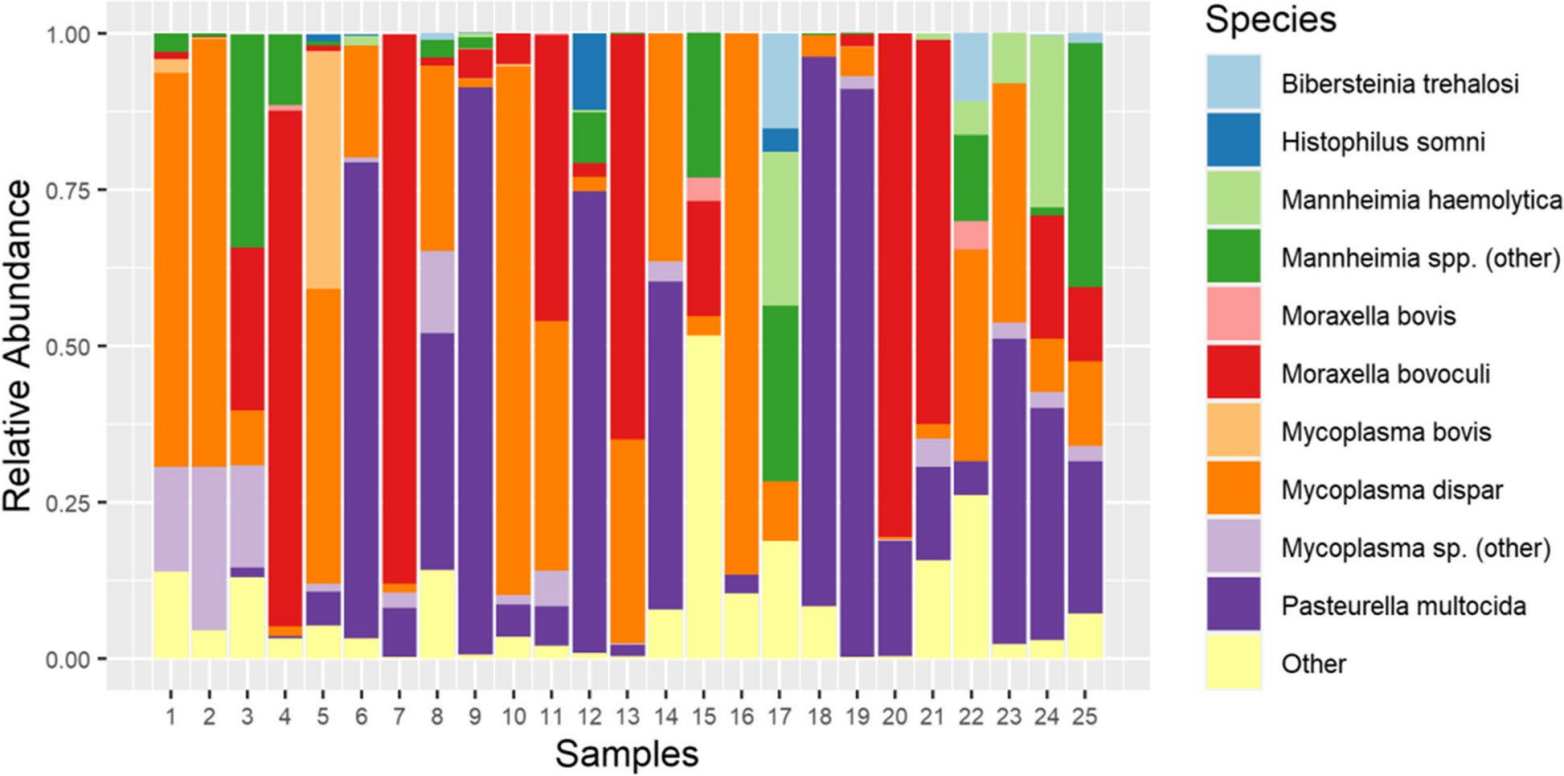
Relative abundance of bacterial taxa across all samples. Each column represents the relative abundance of non-host sequencing bases attributed to ten taxonomic groups of interest. Bases that were classified as anything other than the ten taxonomic groups listed above were grouped into the “Other” category

**Fig. 2:**
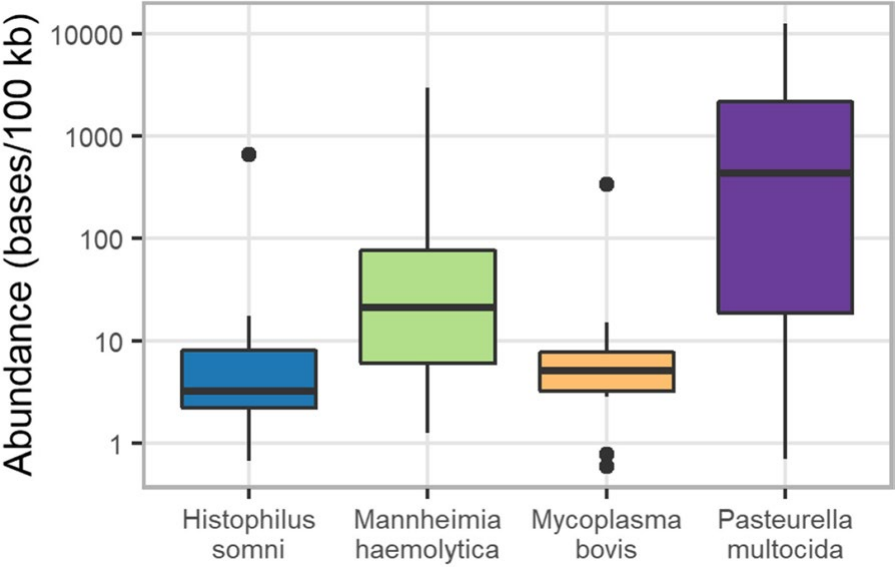
Base-pairs of BRD associated bacteria identified across all samples used in metagenomic sequencing. The number of bases classified as each of the four target organisms was calculated for each sample and expressed per 100 kb sequenced. In each boxplot, the middle horizontal line represents the median, and the top and bottom horizontal line represent the 75th and 25th percentile, respectively. Outlier values are indicated as dots and represent any values that are over 1.5 times the interquartile range over the 75th percentile or under the 25th percentile

*Moraxella bovoculi, M. haemolytica, Mycoplasma dispar,* and *P. multocida* dominated most samples in both the number of reads and the number of bases attributed to these organisms (Fig. 1). *P. multocida* was the most abundant BRD pathogen detected in the metagenomic sequencing data, followed by *M. haemolytica*, *M. bovis*, and *H. somni* (Fig. 2). The only organism detected via metagenomic sequencing in every single sample was *M. dispar*. Regarding the abundance of BRD-associated organisms, *P. multocida* was present in 23 samples, *M. haemolytica* was present in nine samples, *H. somni* was present in seven samples, and *M. bovis* was present in ten samples (Table 2). In general, each sample was dominated by one or two species (typically *M. dispar, M. bovoculi,* or *P. multocida*) with minimal representation from other species (Fig. 1).

**Table 2:** Concordance between culture and sequencing results for each sample

	**Culture Result**				**Number of Sequencing Reads**				**Concordance between Methods**			
*Sample*	*MH*	*PM*	*HS*	*MB*	*MH*	*PM*	*HS*	*MB*	*MH*	*PM*	*HS*	*MB*
1				+				8	MATCH	MATCH	MATCH	MATCH
2				+	6	1	2	10	Seq	Seq	Seq	MATCH
3		Few				9		5	MATCH	MATCH	MATCH	Seq
4						7			MATCH	Seq	MATCH	MATCH
5		1 +		+		49	11	344	MATCH	MATCH	Seq	MATCH
6		2 +			80	4,085	16	1	Seq	MATCH	Seq	Seq
7		1 +		+		1,436	3	1	MATCH	MATCH	Seq	MATCH
8		1 +		+		660		4	MATCH	MATCH	MATCH	MATCH
9		4 +		+	89	14,198	10	8	Seq	MATCH	Seq	MATCH
10		1 +		+		55		4	MATCH	MATCH	MATCH	MATCH
11		1 +		+		209	1		MATCH	MATCH	Seq	Cult
12		3 +		+	24	3,582	593		Seq	MATCH	Seq	Cult
13				+	3	255			Seq	Seq	MATCH	Cult
14		1 +		+		33			MATCH	MATCH	MATCH	Cult
15									MATCH	MATCH	MATCH	MATCH
16						2			MATCH	Seq	MATCH	MATCH
17	1 +				324	3	48		MATCH	Seq	Seq	MATCH
18		2 +				813			MATCH	MATCH	MATCH	MATCH
19		4 +			1	5,739	1	6	Seq	MATCH	Seq	Seq
20		1 +				1,339	1		MATCH	MATCH	Seq	MATCH
21					34	520	1		Seq	Seq	Seq	MATCH
22					65	67			Seq	Seq	MATCH	MATCH
23		1 +			381	2,306		4	Seq	MATCH	MATCH	Seq
24	1 +	1 +			7,926	10,712	9	15	MATCH	MATCH	Seq	Seq
25		1 +		+	27	1,222		5	Seq	MATCH	MATCH	MATCH

The columns under “culture result” indicate the semiquantitative abundance of each recovered isolate (*MH Mannheimia haemolytica*, *PM* *Pasteurella multocida*, *HS* *Histophilus somni*, *MB* *Mycoplasma bovis*). The columns under “Number of sequencing reads” indicate the number of metagenomic sequencing reads that were classified as each of the four target pathogens, regardless of length. The columns under “concordance between methods” indicates whether the two approaches yielded the same answer (“MATCH”), Sequencing detected the organism when culture did not (‘Seq”), or culture detected the organism when sequencing did not (“Cult”)

Twenty-six different ARGs encoding resistance to aminoglycosides (*aph(3’)-Ia, aadA31, aph(3'')-Ib, aph(6)-Id*), beta-lactams (*blaBRO, blaCARB, blaROB*), macrolides (*erm35, ermC, mphE*), phenicols (*cmx, floR*), linocosamides (*lunC, lsaB*), tetracyclines (*tet34, tetB, tetH, tetQ, tetW, tetX, tetY*), and trimethoprim (*dfrA14*) were detected in the metagenomic sequences across 13 unique samples. Genes conferring resistance to the beta-lactam drug class were the most commonly detected and were found in eight different samples. *tetH* was the most frequently detected resistance gene overall (*n* = 6) followed by *mphE* (*n* = 5), *blaROB-5* and *aadA31* (*n* = 4). In 4 of the 8 samples where neither *P. multocida* nor *M. haemolytica* were isolated via culture, ARGs were detected in the sequence data, while in in the 18 samples where these organisms were detected, 8 samples had ARGs in the sequence data.

Fifteen different ARGs encoding resistance to aminoglycosides (*aph(3’)-Ia, aadA31, aph(3'')-Ib, aph(6)-Id*), beta-lactams (*blaROB*), macrolides (*msrE, mphE*), tetracyclines (*tetH, tetY*), sulfonamides (*sul2*) and trimethoprim (*dfrA14*) were detected via WGS across 10 unique isolates. No ARGs were detected in 8 of 18 isolates (44.4%). *sul2*was the most frequently detected resistance gene overall (*n*=10), followed by *aph(6)-Id*(*n*=7) and *aph(3’)-Ia *(*n*=6).

### Concordance between culture and sequencing

In general, more BRD pathogens were detected via metagenomic sequencing than by culturing (Table 2). For five samples, the presence or absence of all four BRD pathogens as determined by culturing and sequencing were in perfect agreement. The rate of concordance between culture and sequencing was highest for the detection of *P. multocida*, where the two approaches yielded the same result in 72% of samples (Cohen’s κ: 0.27). For the detection of *M. bovis* and *M. haemolytica,* the culture and sequence data yielded the same result in 64% and 60% of samples, respectively (Cohen’s κ: 0.28, 0.17). As *H. somni* was not detected by culture in any sample, Cohen’s κ could not be calculated. For those samples where the two diagnostic approaches did not match, sequencing detected *M. haemolytica, P. multocida,* and *H. somni* when culture did not. *M. bovis* was likewise detected via sequencing in five samples where none was recovered by culture, but the reverse was true for four additional samples where the organism was cultured but no sequencing reads were detected.

Concordance between the AST results for the Gram-negative pathogens and the ARGs detected via metagenomic sequencing was generally low (Table 3). Of the six isolates exhibiting phenotypic resistance to beta-lactams (ampicillin, ceftiofur or penicillin), three samples contained relevant ARGs in the metagenomic sequencing data (*blaCARB-5, blaROB-2,4,5*); conversely, five samples without phenotypic resistance to beta-lactams had ARGs associated with resistance to this drug class. Only one of two isolates that demonstrated resistance to macrolides (gamithromycin and tulathromycin) had macrolide resistance genes (*mphE*), whereas five other samples with no phenotypic resistance to macrolides had detectable resistance genes in the sequence data (*mphE, erm35, ermC*). Similarly, ARGs encoding resistance to aminoglycosides and tetracyclines (*tetH, tetX, tet34*) were detected in only 33% (5/15) and two of three isolates with phenotypic resistance to at least one drug in these classes, respectively. Samples without phenotypic resistance to aminoglycosides (*n* = 2) or tetracycline (*n* = 6) nevertheless had ARGs associated with resistance to these classes.

**Table 3:**
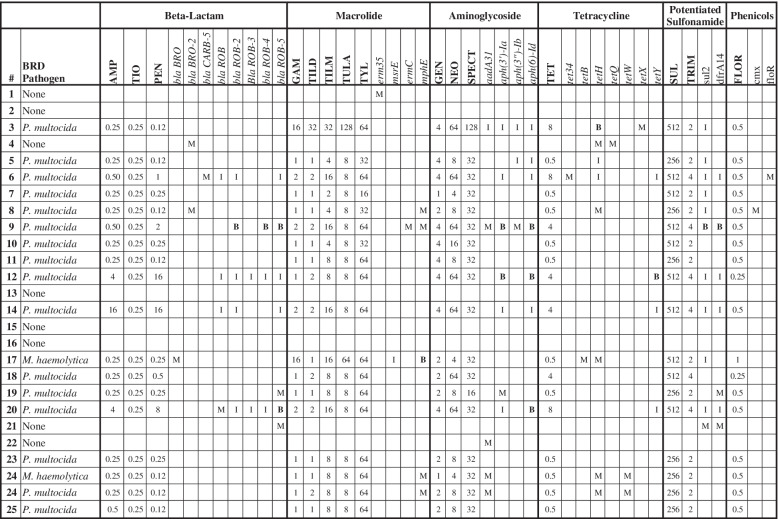
Isolate MICs and associated ARGs recovered from each sample

Each row indicates whether a BRD organism was recovered, the measured MICs for that isolate against the BOPO7F panel, and whether any relevant ARGs were detected in the metagenomic sequencing data. Table boxes with an “M”, “I”, or “B” indicate samples where a certain gene was detected via metagenomic sequencing, WGS, or both methods, respectively

Metagenomic sequencing identified at least one ARG likewise detected by WGS in five of 18 isolates (Table 3). Genes identified by both sequencing methods include *aph(6)-Id* (*n* = 3), *aph(3’)-Ia* and *blaROB* (*n* = 2), and *mphE*, *tetH, tetY, sul2,* and *dfrA14* (*n* = 1). In nine of 18 isolates, metagenomic sequencing identified at least one gene not detected via isolate sequencing; the most frequently detected genes of this type are *mphE* and *tetH* (*n* = 4) and *aadA31* (*n* = 3).

The recovered *M. bovis* isolates were highly resistant to the antimicrobials they were tested against, and elevated MICs were particularly notable for gamithromycin, tildipirosin, tilmicosin and tylosin. WGS revealed that three *M. bovis* isolates had cytosine-to-thymine transitions at positions 683 and 798 of the 23S rRNA gene, which could confer resistance to macrolides. However, several isolates that did not have a SNP at these positions also demonstrated elevated MICs, and therefore agreement between the sequence and AST data was low for this organism.

## Discussion

The upper respiratory tracts of the cattle sampled in this study were dominated by *Moraxella bovoculi, Mycoplasma dispar,* and *Pasteurella multocida*, a finding consistent with other studies of the bovine respiratory microbiome in healthy [24, 25] and unhealthy [26, 27] feedlot cattle in North America. Although *M. bovoculi* is frequently found in the bovine respiratory tract, it is more commonly associated with bovine keratoconjunctivitis [28], though the species from these two niches are genetically distinct [29]. *M. bovoculi* and the broader *Moraxella* genus do not have a recognized role in bovine respiratory disease. In contrast, both *Mycoplasma dispar* and *P. multocida* are not only frequently detected in the nasopharynx as commensal microbiota, but are known opportunistic pathogens that actively contribute to respiratory disease [30–32]. The frequent co-occurrence of these species is remarkable in that several studies suggest synergism between *Mycoplasma* sp. and *P. multocida* [31, 33, 34] as well as a possible initiative role *of M. dispar* in the development of respiratory tract disease in dairy cattle [35–38]. While the pathogenicity of *M. dispar* is well described in dairy calves, particularly in countries free of *M. bovis*, it has received little attention in BRD feedlot studies. However, this could be a consequence of the challenges associated with the fastidious nature of this organism rather than its potential importance in BRD in feedlot cattle [38]. In this regard, culture-independent approaches like metagenomic sequencing offer the opportunity to expand pathogen detection beyond the more typical predetermined BRD pathogens of interest (*M. haemolytica, P. multocida, H. somni*) for which culture-based techniques are well-established.

The detection of *M. bovis* in this study agrees with others examining BRD pathogens associated with chronic pneumonia in feedlot cattle [33, 39, 40]. *M. bovis* is well recognized as causing a caseonecrotic bronchopneumonia that can lead to treatment failure despite repeated antimicrobial therapies [41, 42]. Infection with *M. bovis* has also been associated with a chronic bronchopneumonia and polyarthritis syndrome, a differential diagnosis for some of the cattle within the study’s sample population that displayed concurrent chronic respiratory disease and lameness [39, 43, 44].

In this study, *P. multocida* was the most abundant species in one third of all samples, representing a larger fraction of reads than what has been previously reported in other metagenomic studies on Canadian feedlots. This abundance can be partially explained by the population of chronically diseased animals sampled in this study. Unlike the primary insulting pathogens identified from acutely ill cases of BRD during the early stages of infection, *P. multocida* is an opportunist, and is more often implicated in cattle with subacute or chronic pneumonia [31, 45, 46].

Additionally, while some acute respiratory illnesses are associated with increased nasal shedding of pathogens, as is observed with *M. bovis* [42], it is possible that some of the chronic infections were sequestered within lung lesions, hindering detection via upper respiratory tract sampling. Animals allocated to chronic pens may also be failing to thrive as a sequela of previous lung infection, and therefore would not be shedding high numbers of BRD pathogens, as opposed to an ongoing infection requiring additional treatments. This is supported by the semiquantitative culture results observed, where several of the culture results were described as having few, rare, or light growth. Lastly, chronic pens sampled in this study include cases of nonresponsive pneumonia as well as lameness. Due to differences in the body systems affected, some variations in the bacteria identified from the nasopharynx of these animals were expected. Different antimicrobial protocols would also have been used to address each condition, which may have further diversified the bacterial results obtained between animals.

The differences seen between the dominant organisms in this study and those of previously published studies could potentially also be due to the relatively limited sequencing coverage of bacterial genomes in these samples. However, it is worth noting that most previous studies exploring the nasal microbiome have relied on sequencing one or two hypervariable regions of the 16S rRNA gene, and oftentimes this approach cannot be reliably used to classify sequences at the species or subspecies level [47].

The ARGs detected in these samples were associated with resistance to drugs commonly used in the cattle industry [1], including macrolides (*erm35, ermC, mphE*), phenicols (*florR, cmx*), and tetracyclines (*tet34, tetB, tetH, tetQ, tetW, tetX, tetY*). While these genes were detectable in the metagenomic sequence data, relatively short fragment sizes and limited coverage in this preliminary proof of concept trial often impeded the classification of ARG-containing reads beyond the level of phylum. The ARGs detected in this study are similar to those of previous DNA-based surveys of the bovine respiratory tract [8, 9, 21] and even those of other bovine microbiome sites [48]. In particular, *tet(H)*, the ARG present in the greatest number of samples, has been detected in integrative and conjugative element (ICE)-containing strains of *M. haemolytica, P. multocida,* and *H. somni* isolated from confirmed BRD cases [49].

The other tetracycline resistance genes (*tet34, tetB, tetQ, tetW, tetX, tetY*) detected in this study were found in fewer samples and with the exception of *tet(B)*, are not known to occur in pathogens of the bovine respiratory system in feedlot cattle. *Tet(B)*, which encodes a tetracycline efflux pump, has been found in *E.coli* isolated from bovine feces [50], feedlot fecal composite samples [51], feedlot wastewater lagoons [52], and *P. multocida* isolates derived from cattle [53]. *Tet(W), tet(Q),* and *tet(X)* have been detected in bronchoalveolar lavage and deep nasopharyngeal swab samples collected from feedlot cattle, but it was not evident that these genes were present in BRD pathogens rather than environmental or commensal bacterial populations [8, 21, 54, 55].

The macrolide phosphotransferase gene, *mphE*, was also detected in several samples. This gene has been found in *M. haemolytica* isolates derived from pneumonic bovine lung tissue [22, 56], and bronchoalveolar lavage samples collected from BRD cattle confirmed to have died of BRD [21]. This gene is frequently detected with *msrE*, as the two occur together in an operon on the ICE, *ICEPmu1*, which has been found in several members of the *Pasteurellaceae* family [56, 57]; *msrE* was not detected via metagenomic sequencing in this study, but was detected via isolate sequencing in a single *M. haemolytica* isolate where *mphE* was identified by both methods. The *aadA31* gene was also detected in several samples and encodes a spectinomycin/ streptomycin adenylyltransferase. This gene has previously been detected in *P. multocida* and *H. somni* recovered from confirmed BRD mortalities where it was located inside a variant of the ICE, ICE*Mh1* [58]. A notable unifying theme among the most abundant ARGs in these samples is that many have been detected on ICE found in BRD-associated pathogens. The presence of these ARGs on ICE could explain why they are present in the higher abundance, but additional sequencing coverage would be necessary to confirm this.

Follow-up sequencing of individual isolates highlights the potential of metagenomic sequencing as a diagnostic tool. While the ARGs in the metagenomic samples could not be resolved beyond the phylum level, isolate sequencing confirmed the presence of several metagenomically-identified resistance genes in the BRD pathogen associated with that sample. ARGs previously linked to ICE in the *Pasteurellaceae* family and detected by both methods include *aph(3’)-Ia*, *aph(6)-Id*, *mphE, tetH*, and *sul2*; *blaROB-2* has been found more recently on a plasmid in *M. haemolytica* [59]*.* The trimethoprim resistance gene *dfrA14* was likewise detected by both methods and previously linked to *H. somni* isolates in [14]. In several instances where isolate sequencing could not link a metagenomically-detected ARG to the particular pathogen (e.g., *mphE* and *aadA31* in sample 9 from Table 3) the presence of the ARG in the nasopharyngeal sample is nevertheless relevant given the potential for its acquisition by a mobile genetic element and/or horizontal transfer to members of the *Pasteurellaeae* family.

Due to the complexity of antimicrobial resistance dynamics, some level of discrepancy between the presence of ARGs and phenotypic expression of resistance in this study was to be expected. Several studies have shown genotype–phenotype concordance rates to vary across antimicrobial drugs, BRD bacteria, genes of interest, testing methods used, history of antimicrobial exposure, animal sampling time point (e.g. time of feedlot arrival, revaccination, or time of BRD diagnosis), and sampling location within the respiratory tract [13, 14, 60, 61]. Some general reasons for genotype–phenotype discordance include alternative mechanisms of phenotypic resistance, potential presence of ARGs not yet identified [14], the potential for resistance genes to be present but inactive [60], and availability of approved breakpoints for each drug-bacterium-host combination [62].

Metagenomic data provides a unique lens on the assessment of AMR within the complex community of commensal and pathogenic bacteria of the respiratory system. Culture dependent methods, such as traditional AST and WGS, are constrained to producing data on the single isolate selected for testing. In contrast, metagenomic data can provide information on resistance elements that may be present within different bacterial species as well as within genetically distinct populations of the same bacterial species.

While interpretation of ARGs in clinical samples requires thoughtful consideration, tools to provide rapid detection of ARGs would further support BRD therapies as well as antimicrobial stewardship and AMR surveillance efforts, either alone or in combination with phenotypic results. This is particularly true for genes encoding resistance to macrolides and tetracyclines, the drug classes commonly chosen for BRD treatment and control in feedlots [1, 63].

Most *M. bovis* isolates recovered in this study had high MICs for gamithromycin, tildipirosin, tilmicosin and tylosin. While WGS demonstrated that some of these isolates had point mutations in the 23S rRNA gene that could be responsible for the resistant phenotype, this genetic pattern was not present in all resistant isolates and differed from the SNP signature of previously reported resistant *M. bovis* isolates in other studies [64, 65]. Antimicrobial resistance in *Mycoplasma* spp. differs from that of the other BRD pathogens, as members of this genus typically achieve resistance through point mutations rather than through the acquisition of ARGs [65, 66]. In this study, *M. bovis* reads were detected in 13 samples, ranging in abundance from 1 to 344 reads; the detection of genetic determinants of resistance in the metagenome was therefore not feasible for *M. bovis*, due to the low sequencing coverage. While the accurate detection of SNPs in metagenomes has been previously documented [67], it requires high sequence coverage of target genomes (typically in excess of 100x) [68].

For *M. haemolytica*, *P. multocida* and *H. somni*, metagenomic sequencing detected the presence of the pathogens more frequently than did culture. Indeed, DNA-based approaches are often better equipped to identify the presence of pathogens, as they can detect bacteria that are growth-inhibited or dead following antimicrobial therapy [69]. A comparison of these methods is more complex for *M. bovis*, insofar that the pathogen was recovered from culture but not the metagenomic sequence data on four occasions. The difference in *M. bovis* detection results between nanopore metagenomic sequencing and microbial culture may be attributed to the methodology behind these tools and their strengths and weaknesses. The direct, unenriched, and non-targeted nature of the samples used for metagenomic sequencing in this study likely inhibited appropriate coverage for *M. bovis*. This contrasts with culture, where selective media and incubation are used, and when performed by experienced laboratories, is considered a sensitive method of *M. bovis* detection.

The detection of *M. bovis* may also be a limitation of nanopore metagenomic sequencing using current methods alone. A recent investigation by Bokma et al*.* using a Bayesian latent class model described a lower sensitivity for identification of *M. bovis* with nanopore sequencing (77.3% [95% credible interval, 57.8 to 92.8%]) as compared to the described method of rapid identification of *M. bovis* by MALDI-TOF MS [RIMM] (93.0% [76.8 to 99.5%]) [70]. The investigation by Bokma et al*.* differed from the present study in two key factors. Firstly, the sample population included calves from farms with previous or ongoing *M. bovis* respiratory disease outbreaks. Second, bronchoalveolar lavage fluid (BALf) samples were used, an anatomic site and diagnostic method that would produce fewer host cells and a more limited expected microbial complexity as compared to a DNP sample. Therefore, in spite of the challenges associated with sampling type and limited sample process that occurred in the present proof of concept study, the detection of *M. bovis* is a meaningful demonstration of the diagnostic potential of nanopore metagenomic sequencing.

## Conclusion

The benefits of metagenomic sequencing compared to traditional laboratory methods are severalfold and include the reduced time from sampling to results; the potential to detect unculturable pathogens, or those missed by routine bacterial culture; and the ability to simultaneously identify genetically encoded determinants of resistance. In this study, metagenomic sequencing detected the BRD pathogens of interest more often than did culture, but there was more limited concordance between phenotypic resistance to antimicrobials and the presence of relevant ARGs. This finding is likely due to limited coverage of the target organisms in response to the overabundance of bovine-derived sequences. As sequencing depth of relevant (i.e., non-host) sequences increases, so too will the coverage of BRD pathogens and their genetic determinants of resistance. This goal can be achieved by focusing future work on the preferential enrichment of target sequences in the following ways: 1) optimization of host DNA depletion protocols to reduce the amount of bovine DNA in samples prior to extraction; 2) use of selective sequencing strategies such as Oxford Nanopore’s adaptive sampling method; and 3) targeted DNA enrichment strategies, including bait capture, to reduce the amount of off-target sequence post-extraction. Increasing target sequencing coverage will be an essential component of improving the reliability of this technology, such that it can be better used for the surveillance of pathogens of interest and genetic determinants of resistance, and to inform diagnostic decisions.

## Methods

### Animals

Twenty-five mixed-breed steers from two chronic pens of a commercial feedlot in Saskatchewan were enrolled in the investigation. The holding capacity of the feedlot was approximately 25,000 cattle. Samples were collected once in the summer of 2020, near time of finishing, with cattle weights nearing 1,400 pounds. The two cohorts were purposely selected due to their history of chronic respiratory disease or lameness that was non-responsive to medical management, with most animals having received two or more antimicrobial treatments.

Processing and management of feedlot cattle was performed using standard industry protocols and included on-arrival vaccination for pathogens associated with BRD and clostridial disease, a pour-on anthelminthic drug, a hormone growth implant, and metaphylactic administration of tulathromycin (Draxxin, Zoetis Inc., Florham Park, New Jersey, USA). Cattle were fed a diet that met the National Research Council requirements for beef cattle throughout the feeding period. Experienced pen checkers observed animals daily for signs of clinical illness. Animals exhibiting such signs were treated following disease-specific antimicrobial protocols.

### Sample collection

Sampling was performed during one visit by one veterinarian experienced in collecting nasal and deep nasopharyngeal swabs. To facilitate sample collection, cattle were restrained in a hydraulic chute with neck extenders placed to stabilize the animal’s head. The nares were wiped clean of debris with single use paper towels. Next, the first of three double-guarded nasopharyngeal swabs were collected. The 79 cm, three-pieced culture swab (Continental Plastic Corp., Delevan, Wisconsin, USA) was advanced into the ventral meatus of the nostril until approximately 2 cm ventral from the medial canthus of the eye. The inner sheath was advanced through the outer guard to allow for the swab tip to be extended and vigorously rotated against the pharyngeal mucosa for at least ten seconds. The swab tip was withdrawn into the guarded sheaths prior to removal from the nostril. The swab tip was then cut and placed in 3 ml of Amies transport medium. The procedure was repeated using alternating nostrils for a total of three times per calf and these deep nasopharyngeal (DNP) samples were pooled into the same vial of media. The samples were placed on ice and transported back to the University of Saskatchewan on the day of collection.

### Sample processing for metagenomic sequencing

The swabs and media were vortexed for one minute to release biomass from the swab into the transport medium. After vortexing, the swabs were removed. Two ml of media were centrifuged at 9,000 × g for five minutes to pellet all biomass, and then 1800 ul of supernatant were decanted to reduce the total volume to 200 ul. The pellet was resuspended in the remaining supernatant and host depleted using the MolYsis Basic5 kit according to manufacturer’s instructions (Molzym, Bremen, Germany). The resulting host depleted biomass pellet was then used in a total nucleic acid extraction with the MasterPure Complete DNA and RNA Purification Kit according to the manufacturer’s instructions (Lucigen, Middleton, Wisconsin, USA). Extracted DNA was quantified using the Qubit™ 1X dsDNA High Sensitivity Assay Kit (Invitrogen, Carlsbad, California, USA) according to manufacturer’s specifications and then kept at 4 °C until library preparation.

### Culture and antimicrobial sensitivity testing

Pooled, vortexed media was processed the same day for the isolation of *Mannheimia haemolytica, Pasteurella multocida, Histophilus somni,* and *Mycoplasma bovis*. One chocolate agar and one blood agar plate were inoculated with 10 µl of sample and incubated at 35°C in CO_2_ for 48 h. At 24 and 48 h, plates were examined for growth and bacterial colonies suspected of being *M. haemolytica, H. somni,* or *P. multocida* were confirmed using a MALDI-TOF MS Microflex LT instrument and MALDI Biotyper software (Bruker Corporation, Billerica, Massachusetts, USA). BRD pathogens isolated from samples were tested for antimicrobial susceptibility via serial broth microdilution using a commercially available panel (BOPO7F). Minimum inhibitory concentrations (MICs) for each antimicrobial were compared against breakpoints designated by the Clinical and Laboratory Standards Institute [71].

The culture of *M. bovis* was performed as described previously [72]. Briefly, 100ul of pooled sample were inoculated onto PPLO (pleuropneumonia-like organisms) broth with 500 U/mL penicillin G prepared in-house by Prairie Diagnostic Services). Broths were incubated at 35°C in a 5% CO_2_ incubator for 48 h. After incubation, a 10ul loop of broth culture was streaked onto a PPLO agar plate (prepared in-house). Plates and broths were then incubated an additional 48 h. On day 5, the PPLO agar plates were microscopically inspected. Colonies exhibiting typical *Mycoplasma* morphology underwent confirmation of species identification using a MALDI-TOF MS Microflex LT instrument and MALDI Biotyper software (Bruker Corporation, Billerica, Massachusetts, USA).

Antimicrobial susceptibility testing (AST) for *M. bovis* was performed using a microdilution assay, customized into a 96-well Sensititre™ plate (Trek Diagnostics, Oakwood, GA, USA) designed by Jelinski et al. [72]. The antimicrobials included: enrofloxacin (ENRO), 0.12–128 g/mL; tildipirosin (TILD), 0.12–128 g/mL; gamithromycin (GAM), 0.25–256 g/mL; tulathromycin (TULA), 0.25–256 g/mL; tildipirosin (TILD), 1–256 g/mL; tylosin tartrate (TYL), 1–128 g/mL; florfenicol (FLOR), 0.25–256 g/mL; oxytetracycline (OXY), 0.5–256 _g/mL; and chlortetracycline (CTET) 1–256 g/mL. Penicillin (PEN) (2–8 g/mL) served as a control. Growth was assessed using a color redox indicator, alamarBlue (Invitrogen, Fisher Scientific). The *M. bovis* reference strain ATCC® 25,523™ was used for quality control.

To begin AST, broth cultures of *M. bovis* isolates were sub-cultured into a neat PPLO broth and incubated for an additional 24 h. The optical density at 450 nm was determined using NanoDrop One Spectrophotometer (Fisher Scientific) and cultures were normalized to an OD_450_ = 0.1. Cultures were further diluted up to 50X in neat PPLO media and the final inoculum included 120ul of diluted culture into 6 ml of 2X alamarBlue. A 50 μl of the inoculum was added to each well using Sensititre AIM Automated inoculation System (Thermofisher Scientific). The plates were sealed with permeable film and incubated at 35°C ± 10C in a 5% CO_2_ for 48–72 h. Minimum inhibitory concentrations were visually determined at 48 and 72 h. If growth was observed in the positive control wells, MIC values for that isolate were accepted.

As there are no approved MIC breakpoint values for *Mycoplasma bovis*, antimicrobial susceptibility for *M. bovis* was assessed according to existing recommendations [72].

DNA was extracted from all recovered isolates for use in WGS. The MasterPure Complete DNA and RNA Purification Kit was used to extract DNA from *M. bovis* isolates according to the manufacturer’s instructions (Lucigen, Middleton, Wisconsin, USA). DNA extraction for *P. multocida* and *H. haemolytica* was performed using the QIAGEN DNeasy Blood & Tissue Kit (QIAGEN, Hilden, Germany).

### Library preparation and sequencing

Extracted sample DNA was prepared for metagenomic sequencing using the Oxford Nanopore SQK-PBK004 kit (Oxford Nanopore, Oxford, England). After end repair and barcode ligation, sample DNA was amplified with LongAmp Hot Start Taq DNA Polymerase (New England Biolabs, Ipswich, Massachusetts, USA) using the following cycling conditions: 3 min. denaturation at 95°C, 14 cycles of denaturation at 95°C for 15 s, annealing at 56°C for 15 s, extension at 65°C for 6 min 40 s, and a final extension step at 65C for 6 min. The final fragment size of each library was determined with a Genomic DNA ScreenTape on a 4150 TapeStation System (Agilent, Santa Clara, California, USA) and then pooled in equimolar concentrations in groups of 4 to 5 samples. Sequencing was performed on a GridION Mk1 for 72 h per run using FLO-MIN106 R9.4.1 flow cells.

Library preparation for BRD pathogen isolates was performed with the ONT ligation kit SQK-LSK109 and native barcoding kits EXP-NBD104 and EXP-NBD114 as per manufacture’s instructions. Barcoded isolate DNA was pooled into one library and quantified with the Qubit HS dsDNA Assay kit. 200 ng of prepared library was loaded onto a FLO-MIN106 flow cell and sequenced on an ONT GridION device for 72 h.

### Bioinformatic analysis of metagenomic sequencing data

Basecalling of raw signal was performed using Guppy (v4.0). After basecalling, terminal adapters and internal adapters in split reads were trimmed with Porechop v0.2.4 [73]. NanoFilt v2.6.0 was then used to remove any reads shorter than 100 bp, and sequence statistics were calculated using NanoStat v1.5.0 [74].

To classify host and non-host reads, Kraken2 v2.0.8-beta [75] was used with a confidence threshold of 0.1 and a custom database. The classification database included all complete genomes in RefSeq for the bacterial, viral, and archaeal domains, and all RefSeq plasmid nucleotide sequences as of October 17, 2020. The *Bos taurus* reference genome assembly ARS-UCD1.2_Btau5.0.1Y, which is available at http://www.1000bullgenomes.com/ [76] was also added to the classification database. Following classification, reads were split into two datasets using the KrakenTools v1.0 utility extract_kraken_reads.py: those assigned to the *B. taurus* taxid 9913 (host dataset), and those that were classified as any other taxid or were unclassified (non-host dataset).

The custom script kmer_filter.py was used to retrieve chimeric reads (an artefactual fusion of *B. taurus* and bacterial sequence) from the host dataset. This script detects reads where < 75% of the Kraken2-defined *k*-mers are not classified as *B. taurus* relative to the total number of *k*-mers, excluding *k*-mers classified as root (taxid 1), cellular organisms (taxid 131,567), or unclassified (taxid 0). Reads that meet this criterion were then removed from the host dataset and added back to the non-host dataset in order to allow for possible cases of chimeric data (host + microbial). NanoStat was used to generate sequence statistics for the non-host dataset.

Bracken v2.5 [77] was used to generate taxonomic abundance estimates using the output of Kraken2. Bracken was run with default options, and reads assigned to taxid 9913 (i.e., potentially chimeric reads) were filtered using the KrakenTools script filter_bracken.out.py. Krona v2.7.1 [78] was used to create interactive Krona-style plots for visualization of the Kraken2 results.

Both Abricate v1.0.1 [79] and AMRFinderPlus v3.9.8 [80] were used to identify ARGs in the non-host reads using the NCBI Bacterial Antimicrobial Resistance Reference Gene Database (PRJNA313047, version 2020–12-17). The minimum percent identity and percent coverage for ARG detection by Abricate was kept at the default value of 80% for both, while a lower threshold of 60% was used for AMRFinderPlus, as AMRFinderPlus tends to be more stringent in reporting ARGs. The *-plus* option was also used with AMRFinderPlus, which directs the program to also search for genes involved in virulence, biocide, heat, metal, and acid resistance.

For samples where metagenomic contigs could be assembled, Abricate and AMRFinderPlus were also used to detect ARGs in contigs and any unassembled reads, after mapping the non-host reads to the metagenomic contigs with Minimap2 v2.13-r850 [81] (with pre-set input option *map-ont*) and filtering unmapped reads with Samtools v.1.10 [82].

To detect plasmid fragments in the non-host dataset, reads were used as queries in BLASTN searches against the PLSDB plasmid database (v2020_11_19), a curated database of bacterial plasmids from NCBI [83]. BLAST + v2.10.0 [83, 84] was used with an E-value cutoff of 1E-6, a minimum percent identity cutoff of 80%, and a minimum query coverage per HSP cutoff of 80%. Due to the relatively small BLAST database, shorter reads, and high plasmid sequence similarity, the custom script parse_plasmids.py was used to parse the output. This script generates a file with the top hit for each read and taxonomic annotation when the bitscore of the top hit is at least 10 points higher than the next hit from a different organism.

Metagenomic contig assembly of non-host reads was performed with Flye v2.8.1-bl676 [85, 86]. The *-meta* flag was used to indicate metagenome assembly mode, which is designed for highly non-uniform coverage and is sensitive to underrepresented sequence at low (< 2X) coverage. The input reads were specified as *-nano-raw*, which supports ONT reads that have not undergone error correction.

The Contig Annotation Tool (CAT) v5.2.3 [87] was used for contig classification with the 2020–06-18 version of the CAT database. Default parameters were used, with the addition of the *-only-official* flag when running the *add_names* module. This reports only classifications with the standard taxonomic levels (e.g., superkingdom, phylum, etc.), allowing the program to output a summary of the classification results.

The results of the workflow analyses were then parsed by a custom script, summarize_results.py. This script creates an Excel (.xslx) file that summarizes sequence statistics, ARGs, metagenomic assembly statistics, taxonomy results, and plasmid results. It adds further value to the output files generated by the programs in the workflow, as it also calculates and reports the percent non-host sequence and annotates ARGs with Kraken2 and plasmid taxonomy information where appropriate.

### WGS sequence analysis

For sequencing reads derived from *M. haemolytica* and *P. multocida* isolates, read quality control and length filtering were performed as described for the metagenomics data. Abricate v1.0.1 [79] was used to detect ARGs using the NCBI Bacterial Antimicrobial Resistance Reference Gene Database (PRJNA313047, version 2020–12-17). The minimum percent identity and percent coverage for ARG detection by Abricate was kept at the default value of 80% for both.

WGS sequencing data derived from *M. bovis* isolates was assembled using Flye Assembler v2.8.1-bl676 [85]. Final consensus sequences were generated after polishing by Medaka (v.0.10.0; ONT). The presence of single nucleotide polymorphisms (SNPs) in *M. bovis* genomes conferring antimicrobial resistance were predicted by Snippy (v.2.6) [88].

## Supplementary Information

**Additional file 1: Supplementary Table 1.** Sequencing output and post analysis yield across all samples. Post QC statistics refer to the reads that remained after adapter and barcode removal and quality trimming. Non-host statistics and post-host filtering statistics describe the fraction of reads and bases that were not classified as *Bos Taurus* by the bioinformatic pipeline.

**Additional file 2: Supplementary Table 2.** Antimicrobial susceptibility breakpoints for *Mannheimia haemolytica*, *Pasteurella multocida*, and *Histophilus somni*; BOPO7F plate, 2020

## Data Availability

The datasets generated in this study are available in the NCBI Sequence Read Archive (SRA) under BioProject: PRJNA809384 http://www.ncbi.nlm.nih.gov/bioproject/809384.
