# Supplementary material for: Evaluating the potential of third generation metagenomic sequencing for the detection of BRD pathogens and genetic determinants of antimicrobial resistance in chronically ill feedlot cattle
Source: BMC Vet Res. 2022 Jun 2;18:211. doi: 10.1186/s12917-022-03269-6 (PMC9161498; doi:10.1186/s12917-022-03269-6)
Supplement: Supplementary file 1 — Additional file 1: Supplementary Table 1. Sequencing output and post analysis yield across all samples. Post QC statistics refer to the reads that remained after adapter and barcode removal and quality trimming. Non-host statistics and post-host filtering statistics describe the fraction of reads and bases that were not classified as Bos Taurus by the bioinformatic pipeline. [file 12917_2022_3269_MOESM1_ESM.docx]

|  | **Post-QC Statistics** | | | | | **Non-Host Statistics** | | **Post-Host Filtering Statistics** | | | | |
| --- | --- | --- | --- | --- | --- | --- | --- | --- | --- | --- | --- | --- |
| **Sample No.** | **Total bases** | **Number of reads** | **Mean read length** | **Median read length** | **Median read quality** | **% non-host sequence** | **% non-host reads** | **Total bases** | **Number of reads** | **Mean read length** | **Median read length** | **Median read quality** |
| 1 | 3705372994 | 2897083 | 1279 | 1192 | 14.3 | 4.4 | 4.8 | 162037576 | 138904 | 1167 | 1102 | 8.9 |
| 2 | 1466605354 | 1330412 | 1102 | 963 | 13.8 | 5.2 | 5.8 | 76019675 | 76708 | 991 | 814 | 9.4 |
| 3 | 2534541136 | 1758397 | 1441 | 1295 | 13.8 | 6.3 | 6.8 | 159283671 | 119594 | 1332 | 1175 | 8.8 |
| 4 | 1337149775 | 1391251 | 961 | 880 | 13.8 | 6.8 | 7.1 | 90686511 | 98154 | 924 | 836 | 9.0 |
| 5 | 1539340623 | 1697268 | 907 | 804 | 13.6 | 6.4 | 7.3 | 98534303 | 123408 | 798 | 689 | 9.0 |
| 6 | 1457601248 | 1733590 | 841 | 733 | 13.9 | 7.4 | 7.9 | 108380221 | 136523 | 794 | 683 | 9.1 |
| 7 | 2452298283 | 1644370 | 1491 | 1321 | 13.8 | 7.2 | 7.4 | 177134742 | 122052 | 1451 | 1261 | 9.0 |
| 8 | 2675788701 | 2584887 | 1035 | 947 | 14.3 | 5.0 | 5.5 | 133755257 | 140977 | 949 | 874 | 9.1 |
| 9 | 2224048903 | 1978129 | 1124 | 968 | 13.7 | 6.7 | 7.1 | 147832625 | 141133 | 1048 | 895 | 9.2 |
| 10 | 1713805000 | 2012667 | 852 | 801 | 14.4 | 5.0 | 5.5 | 86128686 | 110085 | 782 | 745 | 9.2 |
| 11 | 2154480683 | 2369476 | 909 | 906 | 13.6 | 6.6 | 7.6 | 142282091 | 179400 | 793 | 752 | 9.2 |
| 12 | 1621310658 | 1808894 | 896 | 816 | 14.2 | 5.3 | 5.9 | 85108446 | 106001 | 803 | 720 | 9.1 |
| 13 | 1721092298 | 2220332 | 775 | 682 | 14.0 | 7.6 | 7.9 | 130612067 | 176212 | 741 | 636 | 9.2 |
| 14 | 1739226766 | 1898794 | 916 | 808 | 14.4 | 4.6 | 5.2 | 79227206 | 98388 | 805 | 708 | 8.9 |
| 15 | 1454790362 | 1854989 | 784 | 711 | 14.2 | 5.4 | 6.1 | 78772049 | 112585 | 700 | 624 | 9.1 |
| 16 | 1681506333 | 1655067 | 1016 | 933 | 14.3 | 4.6 | 5.3 | 77866781 | 87808 | 887 | 808 | 8.9 |
| 17 | 1397275012 | 1813173 | 771 | 704 | 14.4 | 5.5 | 6.0 | 77436394 | 108396 | 714 | 644 | 9.2 |
| 18 | 1923408099 | 1967788 | 977 | 908 | 14.4 | 5.0 | 5.5 | 96482653 | 107338 | 899 | 844 | 9.0 |
| 19 | 1509378358 | 1277277 | 1182 | 1039 | 13.8 | 4.6 | 5.3 | 68862228 | 67067 | 1027 | 872 | 9.3 |
| 20 | 1097488200 | 1205465 | 910 | 811 | 13.8 | 5.1 | 5.7 | 56442676 | 68261 | 827 | 711 | 9.4 |
| 21 | 1097385877 | 1306404 | 840 | 746 | 13.9 | 5.1 | 5.8 | 56176363 | 75446 | 745 | 642 | 9.4 |
| 22 | 1403758463 | 1640438 | 856 | 788 | 13.7 | 7.9 | 8.4 | 110807651 | 136896 | 809 | 749 | 9.0 |
| 23 | 1935507520 | 1818927 | 1064 | 1027 | 13.7 | 6.5 | 7.0 | 125984950 | 127286 | 990 | 968 | 8.9 |
| 24 | 2909555883 | 3122872 | 932 | 821 | 13.7 | 8.4 | 8.4 | 243835385 | 262914 | 927 | 772 | 9.5 |
| 25 | 3384396802 | 3390508 | 998 | 880 | 13.7 | 6.4 | 6.7 | 215121018 | 228241 | 943 | 797 | 9.1 |

**Supplementary Table 1. Sequencing output and post analysis yield across all samples.** Post QC statistics refer to the reads that remained after adapter and barcode removal and quality trimming. Non-host statistics and post-host filtering statistics describe the fraction of reads and bases that were not classified as *Bos Taurus* by the bioinformatic pipeline.
