# Supplementary material for: Evaluating the potential of third generation metagenomic sequencing for the detection of BRD pathogens and genetic determinants of antimicrobial resistance in chronically ill feedlot cattle
Source: BMC Vet Res. 2022 Jun 2;18:211. doi: 10.1186/s12917-022-03269-6 (PMC9161498; doi:10.1186/s12917-022-03269-6)
Supplement: Supplementary file 2 — Additional file 2: Supplementary Table 2. Antimicrobial susceptibility breakpoints for Mannheimia haemolytica, Pasteurella multocida, and Histophilus somni; BOPO7F plate, 2020 [file 12917_2022_3269_MOESM2_ESM.docx]

| **Supplementary Table 2.** Antimicrobial susceptibility breakpoints for *Mannheimia haemolytica*, *Pasteurella multocida*, and *Histophilus somni*; BOPO7F plate, 2020 | | | | | |
| --- | --- | --- | --- | --- | --- |
|  | **Antimicrobial** | **Range tested (µg/mL)** | **Breakpoints^a^ (µg/mL)** | | |
|  |  |  | **S** | **I** | **R** |
|  | Ceftiofur | 0.25 - 8 | ≤ 2 | 4 | ≥ 8 |
| **I** | Danofloxacin^b^ | 0.12 - 1 | ≤ 0.25 | 0.5 | ≥ 1 |
|  | Enrofloxacin | 0.12 - 2 | ≤ 0.25 | 0.5 - 1 | ≥ 2 |
|  | Ampicillin | 0.25 - 16 | ≤ 0.03 | 0.06 - 0.12 | ≥ 0.25 |
|  | Clindamycin | 0.25 - 16 | N/A | N/A | N/A |
|  | Gamithromycin | 1 - 8 | ≤ 4 | 8 | ≥ 16 |
|  | Gentamicin | 1 - 16 | N/A | N/A | N/A |
|  | Neomycin | 4 - 32 | N/A | N/A | N/A |
| **II** | Penicillin | 0.12 - 8 | ≤ 0.25 | 0.5 | ≥ 1 |
|  | Spectinomycin | 8 - 64 | ≤ 32 | 64 | ≥ 128 |
|  | Tiamulin | 0.5 - 32 | N/A | N/A | N/A |
|  | Tildipirosin | 1 - 16 |  |  |  |
|  | *M. haemolytica* |  | ≤ 4 | 8 | ≥ 16 |
|  | *P. multocida and H. somni* |  | ≤ 8 | 16 | ≥ 32 |
|  | Tilmicosin^c^ | 2 - 16 | ≤ 8 | 16 | ≥ 32 |
|  | Trimethoprim- | 2/38 | N/A | N/A | N/A |
|  | sulfamethoxazole |  |  |  |  |
|  | Tulathromycin | 8 - 64 | ≤ 16 | 32 | ≥ 64 |
|  | Tylosin tartrate | 0.5 - 32 | N/A | N/A | N/A |
| **III** | Florfenicol | 0.25 - 8 | ≤ 2 | 4 | ≥ 8 |
|  | Sulfadimethoxine | 256 | N/A | N/A | N/A |
|  | Tetracycline | 0.5 - 8 | ≤ 2 | 4 | ≥ 8 |
| Roman numerals I to III indicate the ranking of antimicrobials based on importance in human medicine as defined by the Veterinary Drugs Directorate  S = susceptible, I = intermediate susceptibility, R = resistant  ^a^Breakpoints as defined in CLSI VET01S ED5:2020, *Performance Standards for Antimicrobial Disk and Dilution Susceptibility Tests for Bacteria Isolated From Animals, 5^th^ edition.* No CLSI or other interpretative criteria were available for clindamycin, gentamicin, neomycin, tiamulin, trimethoprim-sulfamethoxazole, tylosin tartrate or sulfadimethoxine.  ^b^Breakpoints are specific to *Mannheimia haemolytica* and *Pasteurella multocida* only; data not available for *Histophilus somni.*  ^c^Breakpoints are specific to *Mannheimia haemolytica* only; data not available for *Pasteurella multocida* and *Histophilus somni.* | | | | | |
|  | | | | | |

| **Antimicrobial susceptibility breakpoints for *Mycoplasma bovis*; custom plate described by Murray Jelinski et al., 2020^a^** | | | | | |
| --- | --- | --- | --- | --- | --- |
|  | **Antimicrobial** | **Range tested (µg/mL)** | **Breakpoints (µg/mL)** | | |
|  |  |  | **S** | **I** | **R** |
| **I** | Enrofloxacin^b^ | 0.12 - 128 | ≤ 0.25 | 0.5 - 1 | ≥ 2 |
| **II** | Gamithromycin^b^ | 0.25 - 256 | ≤ 4 | 8 | ≥ 16 |
|  | Tildipirosin^c^ | 0.12 - 128 | ≤ 4 | 8 | ≥ 16 |
|  | Tilmicosin^c^ | 1 - 256 | ≤ 8 | 16 | ≥ 32 |
|  | Tulathromycin^b^ | 0.25 - 256 | ≤ 16 | 32 | ≥ 64 |
|  | Tylosin tartrate^e^ | 1 - 128 | ≤ 4 | N/A | ≥ 8 |
| **III** | Chlortetracycline^d^ | 1 - 256 | ≤ 2 | 4 | ≥ 8 |
|  | Florfenicol^b^ | 0.25 - 256 | ≤ 2 | 4 | ≥ 8 |
|  | Oxytetracycline^b^ | 0.5 - 256 | ≤ 2 | 4 | ≥ 8 |
| Roman numerals I to III indicate the ranking of antimicrobials based on importance in human medicine as defined by the Veterinary Drugs Directorate  S = susceptible, I = intermediate susceptibility, R = resistant  ^a^Custom plate and associated breakpoints described here: <https://www.mdpi.com/2076-2607/8/1/124/htm>  ^b^There are no CLSI-approved breakpoints for *Mycoplasma bovis.* Breakpoints are adapted from those approved for *Mannheimia haemolytica, Pasteurella multocida* and *Histophilus somni* in CLSI VET01S ED5:2020.  ^c^Breakpoints are adapted from those approved for *Mannheimia haemolytica* only.  ^d^Oxytetracycline breakpoints for *Mannheimia haemolytica, Pasteurella multocida* and *Histophilus somni* were also applied to chlortetracycline.  ^e^Breakpoints for tylosin tartate were derived from MIC data in combination with the presence of single point mutations in one or more alleles. | | | | | |
|  | | | | | |
